# High serum PD‐L1 level is a poor prognostic biomarker in surgically treated esophageal cancer

**DOI:** 10.1002/cam4.2789

**Published:** 2019-12-21

**Authors:** Masaaki Ito, Satoshi Yajima, Takashi Suzuki, Yoko Oshima, Tatsuki Nanami, Makoto Sumazaki, Fumiaki Shiratori, Kimihiko Funahashi, Naobumi Tochigi, Hideaki Shimada

**Affiliations:** ^1^ Department of Clinical Oncology Toho University Graduate School of Medicine Tokyo Japan; ^2^ Department of Gastroenterological Surgery Toho University School of Medicine Tokyo Japan; ^3^ Department of Surgical Pathology Toho University School of Medicine Tokyo Japan

**Keywords:** biomarker, esophageal cancer, overall survival, serum PD‐L1

## Abstract

**Background:**

Programmed death ligand 1 (PD‐L1) inhibitor has been approved as one of the standard therapies for various cancers. Some reports have shown that serum PD‐L1 level is associated with advanced tumor stages and poor prognosis; however, corresponding pathological information in esophageal cancer patients is lacking. Therefore, we evaluated the clinicopathological and prognostic impact of serum PD‐L1 levels in surgically treated esophageal cancer.

**Methods:**

A total of 150 patients who underwent radical resection for esophageal cancer were included in the study. Preoperative serum PD‐L1 levels were analyzed using the enzyme‐linked immunosorbent assay kit. A cutoff level of 65.6 pg/mL was used to divide the patients into two groups, and univariate and multivariate analyses were conducted to compare the clinicopathological characteristics and prognoses between these two groups.

**Results:**

Although significant associations between serum PD‐L1 levels and clinicopathological variables were observed, serum PD‐L1 level was significantly associated with high neutrophil counts, high CRP levels, low albumin levels, and high squamous cell carcinoma antigen levels. Furthermore, serum PD‐L1 level was associated with poor overall survival independent to TNM factors.

**Conclusions:**

High preoperative level of serum PD‐L1 is a prognostic factor for poor overall survival in patients with surgically treated esophageal cancer.

## Graphical abstract

High preoperative level of serum PD‐L1 is a prognostic factor for poor overall survival in patients with esophageal cancer.

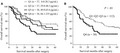

## 1 INTRODUCTION

Despite the development of several multidisciplinary diagnosis/therapies, including surgery, radiotherapy, chemotherapy, and molecular approaches,^1^ esophageal cancer continues to remain as one of the worst malignant neoplasms of the digestive system.^2^ Recently, immune checkpoint inhibitors for advanced esophageal cancer were introduced as a new therapeutic agent.^3^, ^4^ Among them, the expression of tissue programmed death ligand 1 (PD‐L1) was assessed; however, an assessment of serum PD‐L1 levels to evaluate the clinicopathological characteristics of the patients with esophageal cancer has not been made so far.

Recently, several reports have shown that high serum PD‐L1 level is a prognostic factor for poor overall survival in patients with gastric cancer,^5^, ^6^, ^7^ hepatocellular carcinoma,^8^ and biliary tract cancer.^9^ Akutsu et al reported the clinicopathological significance of serum PD‐L1 levels in esophageal cancer patients.^10^ However, their report included only 26 surgically treated cases, other cases were 20 endoscopic submucosal dissection treated cases, and 39 nonsurgical treated cases. Furthermore, Weber et al reported that serum PD‐L1 mRNA expression was a prognostic marker in patients with oral squamous cell carcinoma.^11^ However, they did not evaluate the relationship with other tumor markers, laboratory data, and pathological factors in resected specimens.

Tumor PD‐L1 expression and/or serum PD‐L1 levels have been associated with inflammatory biomarkers, such as high white blood cell count,^7^ CRP levels,^5^ and IL‐6 expression in various types of tumors.^12^ To the best of our knowledge, correlations between serum PD‐L1 levels and these inflammatory biomarkers in patients with esophageal cancer have not been documented so far.

Therefore, in this present study, we evaluated the clinicopathological and prognostic significance of serum PD‐L1 levels in surgically treated esophageal cancer patients. Additionally, we evaluated the relationship among laboratory parameters, inflammatory markers, and serum PD‐L1 levels.

## 2 MATERIALS AND METHODS

### 2.1 Study groups

A total of 150 patients with esophageal cancer who underwent radical surgery at the Toho University Hospital between January 2010 and October 2017 were included in this study. Among them, 87 patients received neoadjuvant chemotherapy. The number of patients in each stage according to the Japanese Classification of Esophageal Cancer (11th Edition^13^) is as follows: stage 0, 11; stage I, 26; stage II, 41; stage III, 57; and stage IVa, 15. All patients were regularly followed up until July 2018 or death. None of the patients had received anti‐PD‐1 or anti‐PD‐L1 antibody treatment. Written informed consent was obtained from all the patients, and the study was approved by the Ethics Committee of the Toho University School of Medicine (nos. A18103).

### 2.2 Serum sampling and enzyme immunoassay for serum markers

Serum samples were collected prior to treatment and stored at −80°C until the assays were performed. Expression levels of serum p‐53 antibodies (p53‐Abs)^14^ and squamous cell carcinoma antigen (SCC‐Ag)^15^ were evaluated. The cutoff values for serum p53‐Abs and SCC‐Ag were fixed at 1.3 IU/ml and 1.5 ng/ml, respectively. Serum PD‐L1 levels were measured using a commercially available enzyme‐linked immunosorbent assay kit for PD‐L1 (R&D Systems, Minneapolis, MN, USA). This assay employs the quantitative sandwich enzyme immunoassay technique using a monoclonal antibody specific for human B7‐H1 pre‐coated onto a microplate. We determined the serum PD‐L1 levels in the collected samples according to the manufacturer's protocol.^5^ We evaluated the neutrophil‐to‐lymphocyte ratio (NLR) and platelet‐to‐lymphocyte ratio (PLR) with serum PD‐L1 level. The cut off level of NLR and PLR was set as follows NLR; 1.6, PLR; 117.07 according to previous reports.^16^, ^17^

### 2.3 Statistical analysis

Continuous data are expressed as mean ± standard deviation. Comparisons between continuous data were carried out using the Mann‐Whitney U test. Differences in the distribution of two variables were evaluated using Fisher's exact test or χ^2^ test and the corresponding differences among three variables using Kruskal‐Wallis test. The association between clinicopathological data and serum PD‐L1 levels was analyzed using logistic regression analysis. Survival curves were calculated using the Kaplan‐Meier method and compared using the log‐rank test. Significant predictors were assessed by multivariate analysis using the Cox proportional hazards model. All analyses were performed using the EZR software.^18^ Statistical significance levels were defined as *P* < .05.

## 3 RESULTS

### 3.1 Serum PD‐L1 level according to the TNM stage

**Figure 1:**
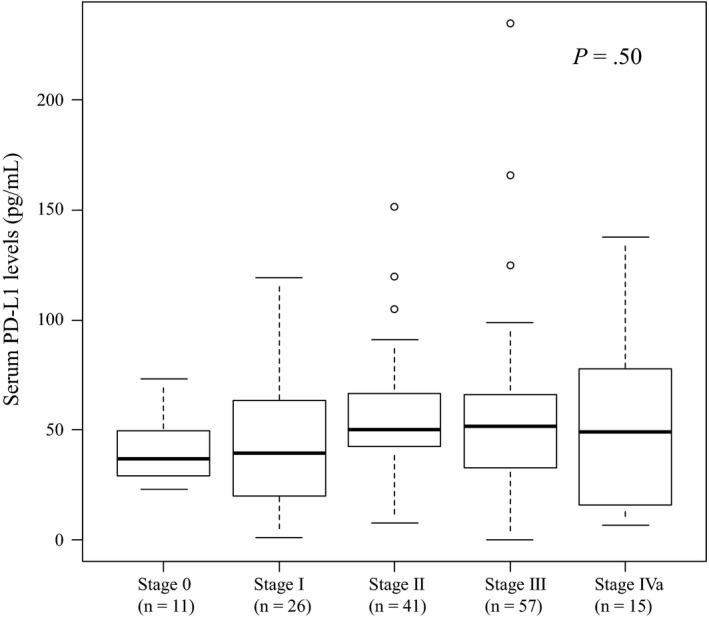
Comparison of serum PD‐L1 levels according to each stage by Japanese Classification of Esophageal cancer. Statistical analysis was performed by the Kruskal‐Wallis test

The mean levels of serum PD‐L1 at each stage were as follows: 37.4 ± 12.2 pg/ml (stage 0), 45.0 ± 30.3 pg/ml (stage I), 55.6 ± 29.0 pg/ml (stage II), 54.5 ± 38.4 pg/ml (stage III), and 53.3 ± 37.5 pg/ml (stage IV). No significant differences in serum PD‐L1 levels were observed among the five stages (Figure 1).

### 3.2 Comparison of serum PD‐L1 levels according to various laboratory data

As seen in Table 1, serum PD‐L1 levels were significantly correlated with neutrophil counts and CRP, albumin, and SCC‐Ag levels; in addition, correlations were observed among serum PD‐L1 level, advanced‐stage cancer, and lymphocyte counts (statistical significance notwithstanding). Alternatively, the serum PD‐L1 levels were not correlated with tumor depth, lymph node status, WBC and platelet counts, and serum p53 antibody and hemoglobin levels.

**Table 1:** Comparison of serum PD‐L1 levels according to various laboratory data of the patients with esophageal carcinoma

		Number of patients	Median (min‐max)	*P* value^a^
Tumor depth	Tis, T1	53	43.1(1.18‐119.2)	.12
	T2 T3 T4	97	51.5(2.40‐235.1)	
Tumor depth	Tis T1 T2	73	44.8(1.18‐151.7)	.22
	T3 T4	77	51.1(2.40‐235.1)	
Lymph node status	Negative	61	47.0(1.18‐119.9)	.60
	Positive	89	51.1(2.40‐235.1)	
Stage	0 I	37	37.0(1.18‐119.2)	.08
	II III IV	113	52.0(2.40‐235.1)	
WBC(/μl)	>8000	136	45.2 (7.35‐96.6)	.55
	≤8000	14	50.2 (1.18‐235.1)	
Neutrophil (%)	>70	37	59.1 (11.1‐151.7)	**<.01**
	≤70	113	44.6 (0.0‐235.1)	
Lymphocyte (%)	>35	35	43.4 (6.59‐105.0)	.06
	≤35	115	52.3 (0.0‐235.1)	
Hemoglobin (g/dl)^b^	>12	75	44.1 (1.18‐119.2)	.10
	≤12	74	52.0 (6.59‐235.1)	
Platelet	>150 000	128	49.4 (1.18‐235.1)	.80
	≤150 000	12	53.2 (11.4‐151.7)	
CRP(mg/dl)^b^	>0.3	64	55.7 (1.18‐235.1)	**.02**
	≤0.3	81	46.2 (2.40‐119.9)	
Albumin(g/dl)	>3.5	119	46.3 (1.18‐151.7)	**<.01**
	≤3.5	31	63.1 (14.1‐235.1)	
SCC‐Ag(ng/ml)^b^	Negative	92	44.6 (1.18‐235.1)	**<.01**
	Positive	55	58.3 (6.59‐165.8)	
p53‐Abs(U/ml)^b^	Negative	108	47.3 (1.18‐235.1)	.29
	Positive	35	52.0 (11.1‐125.1)	

Bold values indicate significant differences.

a Mann‐Whitney *U* Test.

b Loss value.

### 3.3 Relationship between serum PD‐L1 levels and overall survival

**Figure 2:**
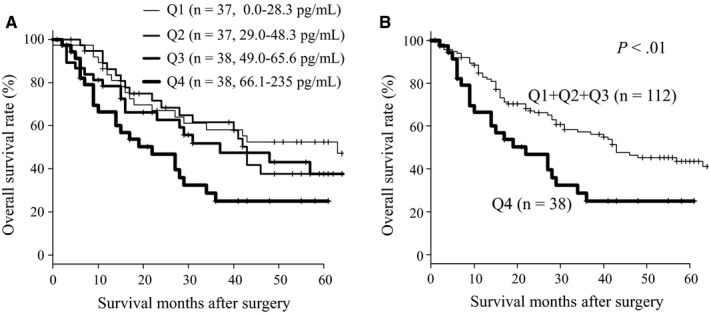
A, Comparison of overall survivals of the patients with esophageal carcinoma according to serum PD‐L1 levels classified into four groups (Q1Q2Q3Q4). B, Comparison of overall survivals according to serum PD‐L1 levels classified into two groups (Q1 + Q2+Q3 vs Q4). Statistical analyses were performed by the Log‐Rank test

In order to clarify the cutoff level of serum PD‐L1, we divided into every one‐fourth into four groups, Q 1, Q 2, Q 3, and Q 4 (Figure 2A). The serum PD‐L1 level of Q1 ranged from 0.0 to 28.3 pg/ml, Q2 ranged from 29.0 to 48.3 pg/ml, Q3 ranged from 49.0 to 65.6 pg/ml and Q4 ranged from 66.1 to 235 pg/ml, and no significant differences in survival were noted among groups Q1, Q2, and Q3. However, Q4 showed significantly worse survival than the other groups (Figure 2B, *P* < .01). Therefore, the cutoff value of serum PD‐L1 was fixed at 65.6 pg/ml in order to compare the clinicopathological factors among Q1, Q2, Q3, and Q4.

### 3.4 Relationship between high serum PD‐L1 level and clinicopathological factors

Serum PD‐L1 levels were significantly associated with SCC‐Ag in the univariate and multivariate analyses (Table 2), but not with gender, age, location, tumor depth, lymph node metastasis, p53 antibody level. There was no statistically significant correlation between NLR or PLR with serum PD‐L1 in the univariate and multivariate analyses.

**Table 2:** Comparison of serum PD‐L1 levels according to clinicopathological characters of the patients with esophageal carcinoma

Variables		Univariate analysis			Multivariate analysis		
		PD‐L1 ≤ 65.6 pg/ml (Q1 + Q2 + Q3)	PD‐L1 > 65.6 pg/ml (Q4)	*P* value^a^	odds ratio	95% CI	*P* value^b^
Gender	Male	90	32	.64			
	Female	22	6				
Age	>65	58	24	.26			
	≤65	54	14				
Location^c^	Upper	16	5	1.00			
	Lower	92	30				
Tumor depth^c^	T1	42	8	.15	0.88	0.30‐2.59	.82
	T2‐T4	66	26				
Lymph node metastasis^c^	N0	48	11	.25	1.50	0.59‐3.82	.40
	N1	61	24				
SCC (ng/ml)^c^	>1.5	31	21	**<.01**	3.93	1.57‐9.83	**<.01**
	≤1.5	75	12				
p53‐Abs (U/ml)^c^	>1.30	24	9	.65			
	≤1.30	79	24				
NLR^c^	≥1.6	74	28	.19			
	<1.6	33	6				
PLR^c^	≥117.07	72	28	.13	1.83	0.66‐5.05	.24
	<117.07	35	6				

Bold values indicate significant differences.

a Fisher's exact probability test.

b Logistic regression analysis.

c Loss value.

### 3.5 Univariate and multivariate analyses of risk factors on survival

In the univariate analysis, patients with high serum PD‐L1 levels showed significantly worse overall survival than those with low serum PD‐L1 levels (Table 3, left panel). Tumor depth, lymph node metastasis, high SCC‐Ag level were also significant prognostic factors. In the multivariate analysis, a high serum PD‐L1 level was significantly associated with poor survival (Table 3, *P* = .04). However, NLR and PLR were not shown significantly worse overall survival.

**Table 3:** Univariate and multivariate analysis of risk factors for overall survival in the 150 patients with esophageal carcinoma

	Univariate analysis *P* value^a^	Multivariate analysis		
		Hazard ratio	95% CI^b^	*P* value^c^
Gender	.08			
Male/ Female				
Age	.36			
>65/ ≤65				
Location	.60			
Upper/Lower				
Tumor depth	**<.01**	2.76	1.49‐5.11	<.01
T1/T2‐4				
Lymph node metastasis	**<.01**	2.30	1.32‐4.01	<.01
N‐/N+				
SCC‐Ag(ng/ml)	**.02**			
>1.5/ ≤1.5				
p53‐Abs(U/ml)	.19			
>1.30/ ≤1.30				
PD‐L1 (pg/ml)	**<.01**	1.70	1.03‐2.80	.04
Q4/ Q1 + Q2 + Q3				
NLR	.63			
≥1.6/<1.6				
PLR	.44			
≥117.07/<117.07				

Bold values indicate significant differences.

a Log‐rank test

b Adjusted 95% confidence interval

c Cox proportional hazard model

## 4 DISCUSSION

Serum PD‐L1 levels were positively correlated with neutrophil counts and CRP and SCC‐Ag levels and negatively correlated with albumin levels in patients with surgically treated esophageal cancer in this study. Although serum PD‐L1 level was not associated with tumor stage, it was an independent risk factor for poor overall survival.

Based on the results of the multivariate analysis, high serum PD‐L1 level was noted as an independent prognostic predictor. One of the explanations for the malignant potential of high serum PD‐L1 levels was that they were the surrogate markers for PD‐L1‐positive tumor cells, which are known to demonstrate high malignant potentials.^19^, ^20^ PD‐L1 is mainly expressed in tumor cells; therefore, high concentrations of PD‐L1 represent large tumor volumes and/or highly malignant tumors.^10^, ^21^ On the other hand, several reports have demonstrated no significant associations between tumoral PD‐L1 expression and serum PD‐L1 levels,^6^, ^22^, ^23^ suggestive of the fact that a high level of serum PD‐L1 might be related to the immunological condition of the patient. Similar to hepatocellular carcinoma^24^ and gastric cancer,^6^ no associations were noted between serum PD‐L1 levels and tumor stage or tumor size. Increased levels of inflammatory cytokines in the serum might contribute to an increase in the levels of serum PD‐L1, but not tissue PD‐L1 expression.

There was no statistically significant correlation in NLR and PLR with high serum PD‐L1 level in Fisher's exact probability test (Table 2). In multivariate analysis, there was not seen positive correlation with PLR in Logistic regression analysis. And NLR did not show tendency to significant even in univariate analysis. When both NLR and PLR were included in the multivariate analysis, there was a possibility of becoming these scores as compound factor with each other in the Logistic regression analysis. Therefore, we decided to put only PLR and excluded NLR.

One of the limitations of this study was that no immunohistochemical analysis was performed to evaluate the impact of tissue protein expression on serum PD‐L1 levels. Serum PD‐L1 may be produced by multiple sources *via* distinct mechanisms from both tumor and immune cells. Several reports have shown that serum PD‐L1 levels might be influenced by inflammatory cytokine induction.^24^, ^25^, ^26^ Likewise, the present study also demonstrated interactions between serum PD‐L1 levels and several inflammatory biomarkers. Secondly, the relationship between serum PD‐L1 levels before and after treatment was not compared in this study. Changes in serum PD‐L1 levels may be an indicator of recurrence or prognosis.^27^ However, studies have reported an increase in serum PD‐L1 levels after radiotherapy in hepatocellular carcinoma^28^ and chemoradiotherapy in rectal cancer.^29^ Perioperative or peritreatment changes in serum PD‐L1 levels in patients with esophageal cancer may have a clinical significance in predicting the treatment response and/or the prognosis. The relationship between serum PD‐L1 level and treatment response to immune checkpoint inhibitors should be evaluated in the future.

## 5 CONCLUSION

Serum PD‐L1 levels were positively correlated with high levels of inflammatory markers and/or SCC‐Ag levels. Despite the lack of any association with tumor stage, serum PD‐L1 level was found to be an independent risk factor for overall poor survival in surgically treated esophageal cancer.

## CONFLICT OF INTEREST

The authors have no conflict of interest to declare.

## Data Availability

The data that support the findings of this study are available from the corresponding author upon reasonable request.
